# Uncovering Microbial Composition in Human Breast Cancer Primary Tumour Tissue Using Transcriptomic RNA-seq

**DOI:** 10.3390/ijms22169058

**Published:** 2021-08-22

**Authors:** Dominik Hadzega, Gabriel Minarik, Marian Karaba, Katarina Kalavska, Juraj Benca, Sona Ciernikova, Tatiana Sedlackova, Petra Nemcova, Martin Bohac, Daniel Pindak, Lubos Klucar, Michal Mego

**Affiliations:** 1 Institute of Molecular Biology, Slovak Academy of Sciences, 845 51 Bratislava, Slovakia; dominik.hadzega@savba.sk; 2 Institute of Molecular Biomedicine, Faculty of Medicine, Comenius University, 813 72 Bratislava, Slovakia; gabriel.minarik@gmail.com (G.M.); michal.mego@nou.sk (M.M.); 3 Department of Oncosurgery, National Cancer Institute, 833 10 Bratislava, Slovakia; marian.karaba@nou.sk (M.K.); juraj.benca@nou.sk (J.B.); martin.bohac@uniba.sk (M.B.); daniel.pindak@nou.sk (D.P.); 4 Translational Research Unit, Faculty of Medicine, Comenius University and National Cancer Institute, 833 10 Bratislava, Slovakia; katarina.hainova@gmail.com; 5 Department of Medicine, St. Elizabeth University, 810 00 Bratislava, Slovakia; 6 Biomedical Research Center of the Slovak Academy of Sciences, Department of Genetics, Cancer Research Institute, 845 05 Bratislava, Slovakia; sona.ciernikova@savba.sk; 7 Comenius University Science Park, Comenius University, 842 15 Bratislava, Slovakia; tatiana.sedlackova@gmail.com; 8 Medirex Inc., 821 04 Bratislava, Slovakia; petra.nemcova@medirexgroup.sk; 9 2nd Department of Oncology, Faculty of Medicine, Comenius University and National Cancer Institute, 833 10 Bratislava, Slovakia

**Keywords:** breast cancer, metatranscriptomics, microbiome, microbiota, Kraken2, primary tumour, circulating tumour cells, RNA-seq

## Abstract

Recent research studies are showing breast tissues as a place where various species of microorganisms can thrive and cannot be considered sterile, as previously thought. We analysed the microbial composition of primary tumour tissue and normal breast tissue and found differences between them and between multiple breast cancer phenotypes. We sequenced the transcriptome of breast tumours and normal tissues (from cancer-free women) of 23 individuals from Slovakia and used bioinformatics tools to uncover differences in the microbial composition of tissues. To analyse our RNA-seq data (rRNA depleted), we used and tested Kraken2 and Metaphlan3 tools. Kraken2 has shown higher reliability for our data. Additionally, we analysed 91 samples obtained from SRA database, originated in China and submitted by Sichuan University. In breast tissue, the most enriched group were *Proteobacteria*, then *Firmicutes* and *Actinobacteria* for both datasets, in Slovak samples also *Bacteroides*, while in Chinese samples *Cyanobacteria* were more frequent. We have observed changes in the microbiome between cancerous and healthy tissues and also different phenotypes of diseases, based on the presence of circulating tumour cells and few other markers.

## 1. Introduction

In recent years it was found that various organisms are inhabiting different parts of the human body. Not only bacteria, but also other forms of life and viruses [1]. It has been estimated that our body harbours 10 times more microbes than our own cells [2]. It was also predicted, that 500–1000 species of bacteria possibly thrive in our body at any one time [3]. In the past, the gut microbiome had the most attention (150 times more genes than in the human genome were found there) [2]. However, bacteria, fungi and viruses have been found in many different places, also in breast tissue or breast tumour tissue [4,5].

As a matter of fact, every organ has its own composition of microbiota. It has been revealed that bacterial composition has an effect on various diseases including metabolic disorders, inflammatory and autoimmune diseases and allergies. Effects on cancer were found at multiple different body parts including the stomach, colon, liver, lung and skin [6]. It was proposed that microbiome contributes to 16–18% off all malignancies [7].

It might be useful to mention, that the terms microbiota and microbiome although easily mixed with each other, have two different meanings. First, microbiota refers to all microbes, counting bacteria, archaea, fungi, viruses and protozoa in a particular environment. Then, the microbiome refers to all genomes of a microbiota and it is often used to describe the entity of microbial traits (functions) encoded by a microbiota [6,8].

Technology of next-generation sequencing allowed to produce amounts of data for genomic and transcriptomic analyses [9]. RNA-seq data from human tissues contain traces of other organisms, in other words, reads that don’t come from human transcripts, but reads originated from transcripts of bacteria, archaea, other eukaryotes or viruses as it was observed in this study and also was observed by various different research groups before [10].

In the time when it was known that microbes can be found in breast milk, Urbaniak et al. decided to look for microbes in normal breast tissue, inspecting 16S rRNA from human samples collected from two different parts of the world. In Canadian samples they found to be most abundant *Bacillus* (11.4%), then *Acinetobacter* (10.0%), *Enterobacteriaceae* (8.3%), *Pseudomonas* (6.5%), *Staphylococcus* (6.5%), *Propionibacterium* (5.8%), *Comamonadaceae* (5.7%), *Gamma-proteobacteria* (5.0%) and *Prevotella* (5.0%). In the Irish samples, the most abundant taxa were *Enterobacteriaceae* (30.8%), *Staphylococcus* (12.7%), *Listeria welshimeri* (12.1%), *Propionibacterium* (10.1%) and *Pseudomonas* (5.3%). [4]. In another study, *Sphingomonas yanoikuyae* was found to be relatively enriched in normal breast tissue [5]. Hieken et al. presented some differences in their results since their dominant phyla were *Bacteroidetes* and they found very little *Proteobacteria* [11]. In other studies, it was reported that healthy breast tissue contains *Prevotella, Lactococcus, Streptococcus, Corynebacterium*, *Micrococcus*, some levels of *Staphylococcus, Bacteroidetes* and *Enterobacteriaceae* [6]. Costantini et al. report, as other studies abundance of *Proteobacteria*, followed by *Firmicutes, Actinobacteria* and *Bacteroidetes* [12].

To summarise the results of published works, it can be concluded that *Proteobacteria* and *Firmicutes* are the most repeatedly reported as dominant phyla in breast tissue [11,12,13]. Their presence in the normal or breast cancer (BC) tissue was suggested to be a result of adaptation to the fatty acid environment and metabolism in the tissue [4].

The origin of the breast microbiome is not entirely clear, but at least part of it might be a result of translocation from the gastrointestinal tract, in addition to the skin, via the nipple-areolar orifices, nipple-oral contact via lactation and/or sexual contact [6,11].

Multiple studies have been reporting differences in microbial compositions between the normal breast tissue and tumour tissue of breast cancer patients [5,10,14,15,16,17]. A higher abundance of bacteria was found in healthy tissue compared to tumour tissue [5]. However, there are some differences in the results of different studies. Normal tissue paired to tumour tissue was found to have different microbial composition compared to normal tissue of healthy women. Interestingly, similar composition of microbes in tumour sites compared to normal paired tissue were reported [14,15]. However, different conclusions have been found in the study of Xuan et al. [5]. Urbaniak et al. report, that compositions of normal adjacent tissue microbiome from women with benign tumours were closer to normal adjacent tissue of women with cancerous tumours than for tissue from healthy subjects. A significant finding is also, that they did not find different microbial profile dependent on stage of tumour or severity/invasiveness of disease. In multiple points this study appears contradictory to the findings of Xuan et al. [14].

It is still being discussed how can possibly microorganisms influence breast cancer progress (if they can). Effects on immune system was proposed as a possible mechanism [5,18]. Microbes can probably also alter some specific pathways and can induce DNA changes [14,17,19,20,21,22]. In addition to the effects on disease itself, the microbiome has also the ability to influence treatment procedure [23,24,25].

Since microbial reads can be found in standard Illumina RNA-sequencing of breast tissue, we used this fact to uncover microbial and viral composition of primary tumour tissue of breast cancer patients and breast tissue of healthy women. In our study we were looking for changes in the microbiome between healthy and cancer tissues and also between different phenotypes of disease: the presence of circulating tumour cells (CTC) in patient’s blood, molecular subtypes of disease and multiple markers presence or absence.

## 2. Results

### 2.1. Normal Breast Tissue Microbial Composition

We analysed RNA-seq total transcriptomic data (rRNA depleted) from primary tumour tissue breast cancer patients and normal tissue from healthy women by Kraken2 and Metaphlan3. Data were acquired from primary breast tumours and normal breast tissue, which were collected from donors from Slovakia. The second dataset, that we used, RNA-seq data originated from primary breast tumour and normal breast tissue collected from donors in China, was acquired from SRA database (SRA study PRJNA553096). Data were trimmed according to quality control results, reads that didn’t map on human genome hg38 (4.7–7.3% of all reads) were used for the identification of microbes. It is necessary to note, we report here the presence of bacterial transcript, what means it is not the numbers of bacteria that lies behind numbers, but it is in fact trail of their activity. Among all sequencing reads (before filtering out human reads) originated from samples collected in Slovakia, 0.029% were assigned to some microbial feature. For Chinese data, we observed a similar number 0.034%. These numbers specify how many microbial transcripts are present in the mix with dominant human transcripts.

In normal breast tissue of healthy Slovak women (5 samples) we observed the presence of 4 predominant phyla of bacteria, led by *Proteobacteria* (47% of total bacteria), while *Bacteroidetes, Firmicutes* and *Actinobacteria* follow (equally 12%). *Hymenobacter* (7%) and *Sphingomonas* (5%) were the most abundant on the level of genus.

**Figure 1:**
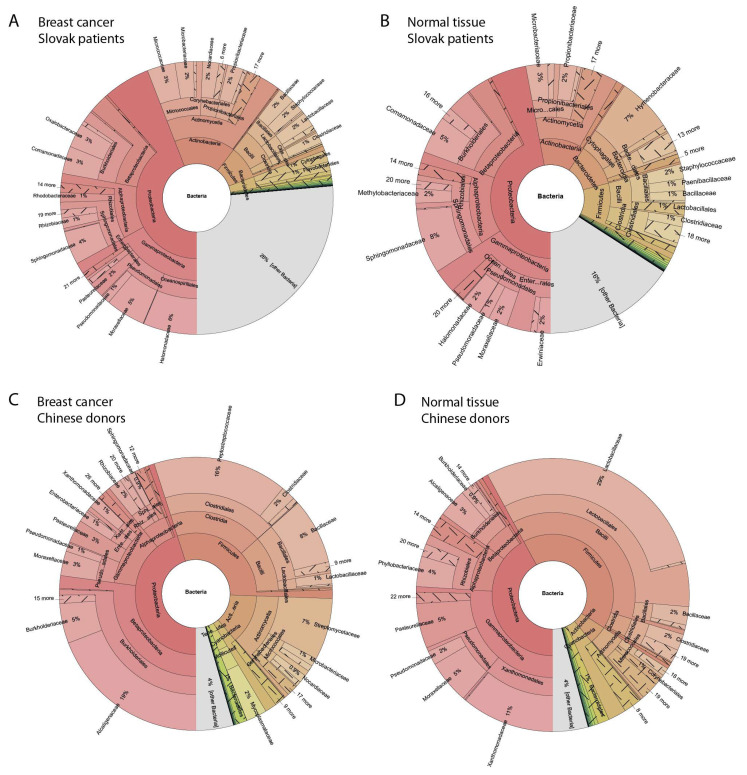
Abundance of different microbial taxa transcript in RNA-seq data. (**A**) in primary tumours of Slovak patients; (**B**) in normal breast tissue of Slovak cancer-free donors; (**C**) in primary tumours of patients from China; (**D**) in normal breast tissue of cancer-free donors from China.

For Chinese samples, the bacterial composition had some differences compared to Slovak data. In normal breast tissue, *Proteobacteria* were a leading force again, with similar abundance as in Slovak samples (42%)*,* but in this case, *Firmicutes* were with the same abundance (42%), making them more prevalent than in Slovak samples and *Actinobacteria* followed (with 5%), while *Bacteroidetes* were significantly less prevalent compared to Slovak samples and were outnumbered by *Cyanobacteria* (4%). The most frequent species were *Lacticaseibacillus rhamonosus* (13%) and *Pasteurella multocida* (5%). Genus *Lacticaseibacillus* was abundant with 25%, *Xanthomonas* 6%. *Sphingomonas*, previously identified as abundant made up just 0.3% of all bacteria and *Hymenobacter* was even more rare. The bundance of the most common bacteria (by transcripts) is shown in Figure 1.

### 2.2. Microbiome in Primary Breast Tumour

In breast tumour tissues of Slovak women (18 samples) the same phyla as in healthy tissue are the most abundant, while a portion is changed: *Proteobacteria* (44%), *Actinobacteria* (16%), *Firmicutes* (9%) and *Bacteroidetes* (3%).

**Figure 2:**
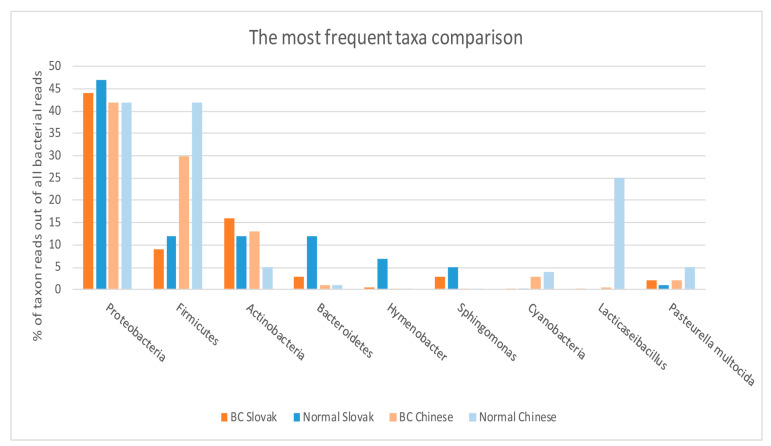
Comparison of abundance (in % of all bacterial reads calculated by Krona evaluation of multiple Kraken2 ouputs) of the most frequent taxa between all basic groups: breast tumour tissue of Slovak patients with breast cancer, normal breast tissue of Slovak donors, breast tumour tissue of Chinese patients with breast cancer (SRA study PRJNA553096), normal breast tissue of Chinese donors (SRA study PRJNA553096), Numerical values for every sample individualy are listed in the Supplementary Table S1.

In breast primary tumours of patients from China, most prevalent were *Proteobacteria* (42%), then *Firmicutes* (30%), *Actinobacteria* (13%), *Cyanobacteria* (3%)*, Tenericutes* (2%) and *Bacteroidetes* (1%). One sample had a rapid increase in *Chloroflexi* (90%), possibly contaminated and left out from complete statistics. Abundance of dominating bacteria in primary breast tumour are shown on Figure 1 and abundances of the most frequently identified taxa (among bacterial transcripts) in tumour tissue and healthy tissue of both geographical origins are compared on Figure 2, which is supplemented by numerical values from individual samples in Table S1 and in Figure S1 illustrating boxplot graphs for all samples.

**Figure 3:**
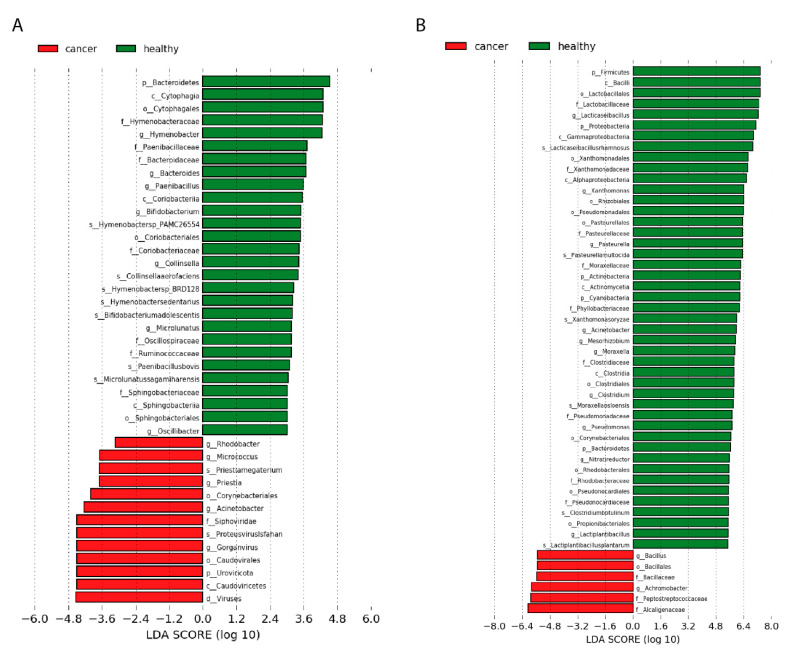
Differentially represented taxa (by different transcript numbers) between normal breast tissue samples (from cancer-free donors) and breast tumour tissue. Taxa on the left have a higher abundance of their transcript in primary breast tumour tissue, while those on the right have higher numbers in normal breast tissue. (**A**) Comparison of the microbiome in primary tumours of Slovak patients and normal breast tissue of Slovak cancer-free donors. Since standard conditions identified too many results, to visualise data, LEfSe was run with parameters (LDA > 3, Kruskal Wallis test *p*-value < 0.05, Wilcoxon test *p*-value < 0.05); (**B**) Comparison of microbiome in primary tumours of 72 patients from China and 18 normal breast tissues of cancer-free donors from China. For visualisation, LEfSe was run with parameters (LDA > 5.5, Kruskal Wallis test *p*-value < 0.05, Wilcoxon test *p*-value < 0.05), for the purpose of visualising the best hits. Full results of LEfSe (LDA > 2) are available on the website http://www.embnet.sk/supp/BC_metatranscriptomics/complete_plots_plotLEfSeResults/ (accessed on 19 August 2021).

In healthy normal tissue of Slovak donors, numerous bacterial taxa were present in higher numbers compared to tumour tissue of Slovak women. Using LEfSe tool for statistical analysis, we report multiple taxa which transcript numbers correlate with disease. The most significantly overrepresented genus in normal tissue was *Hymenobacter* (as it is shown in Figure 3 with other taxa called by LEfSe). In addition, *Bacteroides, Peanibacillus, Bifidobacterium, Pantoea, Collinsella*, *Sphingomonas, Methylobacterium* and multiple other *taxa* were called by LEfSe. The most overrepresented of all taxa were transcripts of phylum *Bacteroidetes*. Most differentially abundant taxa in Slovak samples are also differentially abundant in Chinese, however more taxa were identified to be differentially abundant in Chinese data (with more samples). In the case of Chinese samples, on average 4.6 times more microbial reads (normalised according to total read counts) were found in healthy tissue than in tumour tissue, while in Slovak samples healthy tissues had more microbial transcripts than tumours, although the difference was not as obvious (1.84).

In contrast to many taxa abundant in normal tissue, we identified just a few candidates for overrepresented bacterial transcripts in tumour tissue. The most serious candidate would be *Streptomyces*, although it was found to be in different numbers only in Chinese tissue samples. In both datasets, there were overrepresented viruses *Siphoviridea*, in Chinese patients, also *Myoviridae*. For Slovak patients, genus *Acinetobacter, Rhodobacter, Micrococcus,* order *Corynebacteriales* and species *Priestia megaterium* were enriched in breast tumours. Results are shown in Figure 3 (Filtered more strictly for the purpose of visualisation).

Even though an increasing number of samples from BC patients in case of SRA (Chinese) data analysis (from 19 to 73) did some effect, we got the same bacterial taxa as most significantly overrepresented in normal tissue, while results showed new hits for overrepresented taxa in tumour tissue, however some of them were identified just in few samples (considered outliers) and might be false positives. For example, one extreme sample with a lot of *Chloroflexi* caused this taxon, to be listed as significantly overrepresented in BC.

### 2.3. Association between Clinico-Pathological Characteristics and Microbiome

Additionally, we inspected if there are changes in the microbiome between different phenotypes of breast cancer. Those comparisons were done only for our own samples from Slovakia since we possessed additional information about patients and disease phenotype. This information was not available for Chines samples. Results in the form of graphs, that are not mentioned here, can be found on the website http://www.embnet.sk/supp/BC_metatranscriptomics (accessed on 19 August 2021).

#### 2.3.1. CTC Status (Presence of Circulating Tumour Cells in the Blood)

**Figure 4:**
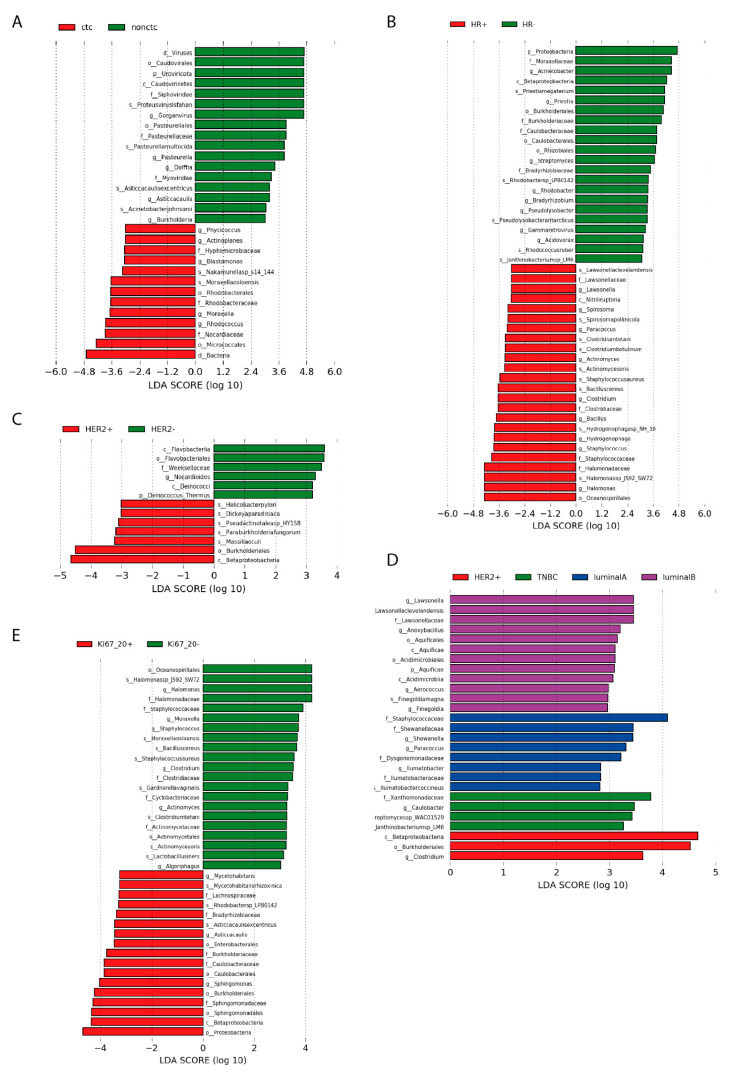
Differentially represented taxa (by different transcript numbers) in primary tumour tissues of Slovak patients between multiple markers statuses. For all comparisons, LEfSe was run with parameters: LDA > 3 (stricter than default LDA > 2 just for purpose of visualisation), Kruskal Wallis test *p*-value < 0.05, Wilcoxon test *p*-value < 0.05. (**A**) Comparison of the microbiome in primary tumours of patients with CTC in their blood and primary tumours of patients without CTC detected in their blood (LDA threshold = 3); (**B**) Comparison of the microbiome in primary tumours of patients positive on HR marker and negative on HR marker; (**C**) Comparison of the microbiome in primary tumours of patients positive on HER2 marker and negative on HER2 marker; (**D**) Comparison of molecular subtypes: Luminal A, B, HER2+, Triple negative (LDA > 2); (**E**) Comparison of the microbiome in primary tumours of Ki67 > 20% and Ki67 < 20%. Full results of LEfSe (LDA > 2) are available on the website http://www.embnet.sk/supp/BC_metatranscriptomics/complete_plots_plotLefSeResults/ (accessed on 19 August 2021).

We looked if there are changes in microbial transcript amounts between patients with circulating tumour cells in their blood and without them. Interestingly there were more groups abundant in tumours of patients with circulating tumour cells (CTC) present in their blood (CTC+). Patients without CTC in their blood (CTC−) were enriched on viruses—order *Caudovirales*: family *Siphoviridae* (genus *Gorganvirus*) and *Myoviridae*. From bacteria, species *Pasteurella multocida* and *Asticcacaulis excentricus* and genus *Delftia*. For CTC+ samples, the most abundant transcripts had *order Micrococcales,* genus *Rhodococcus, Bacillus, Devosia* and *Moraxella (Moraxella osloensis)*. In addition, an abundance of family *Sphingomonadacea, Rhodobacteracea* and order *Rhizobiales* was observed. All taxa correlated with CTC status are shown in Figure 4A. It appears, that tumours of CTC+ patients might be richer for microbiome than tumours of CTC− patients. In tumours of CTC+ patients, 1.88-times more microbial transcript reads (compared to those marked as CTC−) were identified.

#### 2.3.2. Hormone Receptor and HER2 Status

One of the cancer phenotypes comparison, where significant changes in microbial composition were observed was between HR+ and HR− disease. Those patients, which were positive on HR marker have been identified with multiple abundant taxa in their tumour tissues. Worth to mention are genus *Paracoccus*, *Actinomyces, Hydrogenophaga, Halomonas*, species *Cutibacterium granulosum, Bacillus cereus, Staphylococcus aureus, Clostridium tetani, Acinetobacter baumannii* and *Spirosoma Pollinicola*. HR- samples were possibly enriched for genus *Acinetobacter, Rhodobacter* and *Streptomyces*, family *Burkholderiaceae*, species *Priestia megaterium* (Figure 4B). In HER2+ tumours, bacterial group *Burkholderiales* were found to be overrepresented, although only 4 patients had HER2+ disease status (Figure 4C).

#### 2.3.3. P53 Status

For patients positive for p53 protein, some taxa were underrepresented (*Sphingomonas*, *Rhizobiaceae* and species of *Staphylococcus*). Enriched was species *Klebsiella pneumoniae*.

#### 2.3.4. T and N Stage, Tumour Grading

Phyla *Bacteroidetes*, family *Bifidobacteriaceae* (genus *Bifidobacterium*) and bacteria *Clostridium tetani* was associated with smaller tumours (in comparison to tumours smaller than 2 cm and tumours bigger than 2 cm). *Acidobacteria* correlated with a higher T stage.

In the case of axillary lymph node involvement, some microbes are correlated with NO status, which is a group with less advanced disease with a better prognosis. The most possibly enriched are families: *Bacilaceae* (species *Bacillus subtilis*), *Oxalobacteriacea, Microbacteriaceae, Rhizobiaceae, Nocardiaceae, Hymenobacteraceae* (genus *Hymenobacter*) and *Acetobacteraceae*. In the case of tumour grading, a low grade was associated with enrichment of species *Bacillus aureus, Staphyloccocus aureus, Actinomyces oris, Spirosoma Polinica* and also family *Acetobacteraceae*. The high grade was found to be more inhabited by families *Burkholderiaceae, Lachnospiraceae* and species *Pseudolysobacter antarcticus*.

#### 2.3.5. Molecular Subtype

Molecular subtypes of breast cancer were assigned with enriched taxa too. For HER2+ BC, order *Burkholderiales* were enriched compared to other subtypes. From triple negative breast cancer (TNBC) subtype we had just three samples, which were rich for a family *Xanthomonadaceae,* genus *Caulobacter*, species *Janthinobacterium sp_LM6* and *Streptomycessp_WAC01529*. Luminal A subtype was enriched with family *Staphylococcaceae, Dysgonomonadaceae*, genus *Shewanella, Paracoccus* and species *Ilumatobacter coccineus* (found also enriched in some luminal B samples). Luminal B subtype was harbouring transcripts of species *Lawsonella clevelandensis*, order *Aquificales*, genus *Anoxybacillus* (found to be common also in Luminal A). For Luminal A and B subtypes, also species *Finegoldia magna* was specific and plentiful in the number of transcripts (compared to the rest of samples) (Figure 4D).

#### 2.3.6. Proliferation Index Ki67

Multiple taxa correlated with Ki67 proliferation index. Ki67 < 20% tumours were found to be enriched with genus *Halomonas, Moraxella, Staphylococcus, Clostridium* and *Actinomyces* (LDA threshold = 3). The most enriched species with promising enrichment profile were *Bacillus cereus* and *Clostridium tetani*. On the other side, Ki67 > 20% tumours showed to be potentially more inhabited by *Mycetohabitans, Asticcacaulis* and *Sphingomonas* (LDA threshold = 3). Most promising looks *Mycetohabitans rhizoxinica, Rhodobacter sp.* and *Asticcacaulis excentricus* (Figure 4E).

### 2.4. Association of Microbiome with Age

As a control experiment, we used Chinese dataset from SRA and compared healthy donors younger than 50 years old (8 samples) to older donors (10 samples). Donors of age > 50 were identified with more microbial reads in average then younger group (2.25 times more). There were overrepresented taxa found in both groups, although difference between younger and older normal tissue was not as obvious as difference between normal tissue and tumour tissue. In the tissue of older donors, Class *Bacilli*, phylum *Fimicutes* and genus *Lactiseibacillus* were distinctively numerous (LDA < 3.6). In the tissue of younger donors, class *Alphaproteobacteria* genus *Clostridium*, *Praccocus*, Mycobacterium, order *Rhizobiales* and family *Burkholderiaceae*. Graphical visualisation of results for LDA > 2 is available on the website http://www.embnet.sk/supp/BC_metatranscriptomics/complete_plots_plotLefSeResults/LefSe_Nomal_age.png (accessed on 19 August 2021). Complete LEfSe results are available at http://www.embnet.sk/supp/BC_metatranscriptomics/LefSe_plot_differential_features/Normal_age/ (accessed on 19 August 2021).

### 2.5. Presence of Viral Transcripts

**Figure 5:**
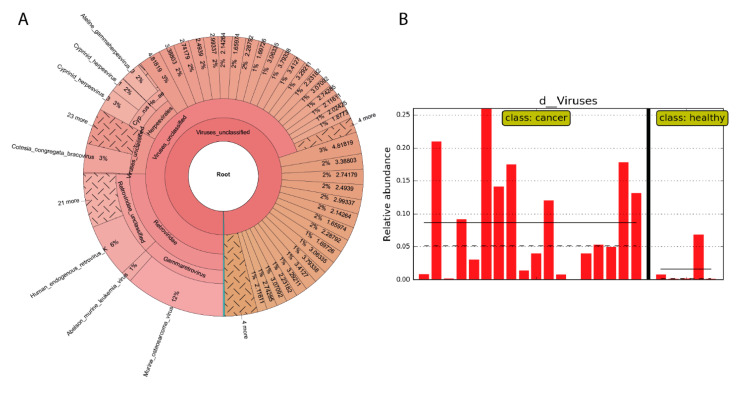
(**A**) Viruses abundance (by Metaphlan3) in breast tissue (both normal and tumour, Slovak donors) visualised by Krona; (**B**) viral transcript numbers identified by Kraken2 in Breast cancer tumours and normal breast tissue of Slovak donors.

To uncover which viruses are present in our samples, we used Metaphlan3, which showed to be more effective in virus identification in training dataset with viruses. The most abundant identified groups of viruses were *Retroviridea, Herpesvirales* and *Bracovirus* (Figure 5A). Except for some hits caused by outliers, we did not observe viruses significantly overrepresented in any group of patients.

Kraken2 was not so sensitive for detection of viruses, but a comparison between tumour tissue and normal tissue shows a tendency of higher amounts of detected viral transcripts in the tumour tissue, as it is shown in Figure 5B.

### 2.6. Pipeline Validation by Artificial Datasets Analysis

To validate our methods and estimate their specificity and sensitivity, we ran additional experiments. As negative control to our analysis, we constructed simulated artificial RNA-seq Illumina reads from human genome hg38 by ART Illumina tool. We made datasets of reads with different lengths, all of them with some errors brought to their sequences to resemble real RNA-seq data. We were observing whether analysis by Kraken2 produced some false positive results. Results were encouraging, since the analysis of reads longer than 40 nt did not produce many false positives (apart from very few cases, insignificant for interpretation of results, bacteria were not called from human-derived reads).

As a positive control we constructed RNA-seq dataset in the same way as previously but from mix of different species of bacteria. We tested if bacteria are correctly identified. Kraken2 performed very well in this experiment and showed very high specificity also on the level of species, although accuracy seemed to degrade towards a more specific taxonomical level. Out of 19 bacteria, just 2 (*Staphylococcus aureus* and *Bacillus subtilis*) were not identified on species level (89.5% sensitivity), while only one read (from 807 classified reads) was called to match taxon that was not present in the synthetic dataset, which was wrongly assigned to another species of bacillus. On the level of genus identification, both specificity and sensitivity were at 100%. The only value that did not appear to be accurate was the comparison of amounts between different species inside of the same genus, which sometimes did not correspond to differences between the number of analysed reads.

Metaphlan3 had worse results for bacteria identification, but still good performance. On the level of species, 13 out of 19 species were accurately identified, while every species was reported on the level of genus. Two species were reported as false positive also on genus level. Ratios of abundancies of simulated transcripts between species were less accurate then Kraken2 abundancies. Different results have been observed for virus identification. Out of 10 viruses, Metaphlan3 identified 7 accurately on the species level, the rest of them were identified on other taxonomic levels (all 3 of them were absent in the database as a species). All false positives were species taxonomically close to true positives and were lower or very low on a number of reads assigned.

## 3. Discussion

In recent years, numerous studies took a challenge to describe the microbial composition of various human tissues [13,26]. While some microbiomes, such as the gut microbiome have been already known to play an important role in human health for a long time [27], other tissues microbiomes have been receiving the attention of scientists more recently. We joined this effort and tried to uncover the microbiome of both, normal and cancerous tissues. While studies of the microbiome are usually done by specialised methods for microbial identification, e.g., 16S rRNA sequencing became standard for this area in previous years, we decided to use our RNA-seq whole-transcriptomic data from our other breast cancer transcriptomic study.

In previous studies, various different methods were applied—identification of microbes using reads from DNA sequencing [10] or microarray (PathoChip) strategy with PCR validation [16,28]. RNA-seq data were used for microbial identification by Thompson et al. [17].

16S rRNA sequencing has been widely used in the research field of metagenomics, also in breast cancer studies [4,5,11,14,15]. 16S rRNA-focused studies only directly characterise the taxonomic profile of a microbiome, but it is a cost-effective option to exhaustively capture the biodiversity of many samples using minimal sequencing [29]. However, this approach has some technical disadvantages. It has been criticised due to a bias in the DNA amplification step of the target gene, inaccuracies in numbers because of the varying number of ribosomal loci across different microbial genomes. The limitation of 16S rRNA gene-based microbial profiling has been partially overcome by the whole shotgun metagenomics (WSM) [30]. Technique for microbial analysis, known as shotgun metagenomics, allows the comprehensive capture of most microbiome members while at the same time elucidating potential genes and functional pathways. However, an important limitation is the inability to distinguish the active from inactive members of a microbiome [29].

And finally, RNA sequencing, compared to the previously mentioned method provides a closer look specifically at active microbial members. With RNA-seq, relatively lowly expressed genes including the entire metatranscriptome that includes non-coding RNAs can be detected, annotated and mapped to metabolic pathways. It has also the possibilities to work with unknown transcripts. Disadvantages are, that sample collection is destructive and sufficient material for sequencing is required (same as in other transcriptomic methods), also metatranscriptomics is not always able to capture the entire metatranscriptome due to the complexity of some microbial communities, the large dynamic range of transcript expression, the short half-life of RNA and a number of technology-specific limitations [29].

The reason why we chose to work with RNA-seq metatranscriptomics, was to test if available methods are able to uncover microbiome and acquire additional information from our RNA-seq data produced for related study [31]. Microbial reads were already known to be present in total RNA-seq data. We tried two pipelines, one based on Kraken2, other on Metaphlan3. The performance of Kraken2 was already found to be sufficient to identify microbes without producing too many false results [32].

By comparing our results to other studies and analysis of training datasets, we can conclude that by RNA-seq analysis it is possible to identify the most prevalent taxa of microbes and it is specific enough to identify organisms on species level as well. We suspected a limit in specificity between close relatives on species level, but our experimental use of Kraken2 tool (Galaxy Version 2.1.1, John Hopkins University, Center for Computational Biology, Baltimore, Maryland, USA) on simulated data with known composition showed encouraging performance on specificity. It appears more limited on sensitivity and accuracy of measuring the number of bacterial reads present, yet it was able to identify most of the bacterial species present in training dataset (89.5%) and all genus. Metaphlan3 did not perform so well on our data, it is probably limited by read lengths. It was able to find most of the genus in training dataset but produced the number of false positives. However, it performed better on the identification of virus species in training dataset compared to Kraken2.

With real data, Metaphlan3 was sensitive with bacteria when settings were set on shorter reads identification, although we detected a high rate of false positives on these settings and potentially false negatives too, so we concluded it is not suited for bacterial detection in our short-read (75 bp) Illumina data. We used Metaphlan3 to detect viruses, although it did not detect significant viral taxa differences between phenotypic classes of cancer.

Interpretation of our findings remains unclear. What is obvious from our data, is that healthy tissue is richer in numerous microorganisms and some groups of microorganisms are potentially enriched in healthy and cancer tissues compared to each other or between different cancer subgroups or phenotypes. However, there is no evidence that any of those microbes in patients of our study influenced the course of disease. It is not obvious if microbes actively join cancerous processes, or their enriched or decreased presence is just an outcome of changed conditions inside of tumour tissue, potentially result of treatment or changed lifestyle. Despite this fact, there is hope for use of information about microbiome composition in favour of breast cancer patients. After all, there is a proof of the impact of the human microbiome on human health and some studies indeed found cancer promoting or protecting abilities of some microbes. For example, in colon cancer, the overabundance of a bacterial species *Fusobacterium nucleatum* correlates with disease and increased likelihood of lymph node metastasis, while *Bacteroidetes fragilis* protects against colitis by modulating inflammatory immune responses in the gut [33,34]. Significant reduction in antibacterial responses was found in breast cancer tumour tissue and that is possibly the logic of bacterial protective effect on breast tissue [5]. *Lactococcus* can activate murine splenic NK cells (which are related to tumour growth) to stimulate cellular immunity [18]. So far, there are multiple suggestions about microbiota having the ability to promote cancer through inducing chronic inflammation, by altering the balance of host cell proliferation and death or by triggering uncontrolled innate and adaptive immune responses [6]. Possible association of several microbes with alterations in the human expression profiles have been reported. For example, *Listeria fleischmannii* showed to be associated with epithelial to mesenchymal transition [17]. The influence of microbiota on oestrogen levels (associated with breast cancer risk) as a way of affecting oncogenesis is also discussed [19] and connection with *Streptococcus* was reported [20,21]. Claims are supported by studies of aspirate fluid of breast cancer survivors [22]. Another way microorganisms might influence breast cancer progress is the ability to induce DNA double-stranded breaks [14].

Microbes seem to influence treatment of disease. For example, gut microbial composition can impact the efficacy of chemotherapy by modulating the translocation, metabolism and immune response to such drugs [23]. Some studies show that an intact microbiome is necessary for optimal responses to anti-cancer therapies [24]. In one study, it was shown, that the microbiome can have an impact on radiotherapy. It is claimed, that bacterial superantigens, specifically *S. aureus*, can exacerbate RT-induced inflammation by further activating T cells and preventing epidermal repair [25]. Microbiome is also suspected to have an effect on immunotherapy efficiency [24].

There is no clear consensus whether or which bacteria, viruses or various microorganisms cause cancer or protect against cancer. Multiple studies present some candidates, which abundance correlate with cancerous changes in breast tissue. In the study done by Borchmann, with dataset of over 3000 samples used to study links between viral and bacterial taxa and cancer, 218 species-level taxa were identified in tumour tissue. Out of these, 27 taxa were judged to be cancer-linked [10].

Multiple taxa and species were reported to be correlated with breast cancer. One of them is *Methylobacterium radiotolerans* was found to be enriched in BC [5] or decreased in cancer patients [15], while *Sphingomonas* *yanoikuyae* showed to be inversely correlated with *M. radiotolerans* in paired normal tissue [5]. Banerjee et al. [16].were studying the presence of different viruses and microbes in different types of breast cancer and found out the microbiome is specific for each of them. In the case of endocrine receptor positive BC, differential signal compared to controls were detected for *Arcanobacterium, Bifidobacterium, Cardiobacterium, Citrobacter, Escherichia*. For triple negative BC, there were differences in *Bordetella, Campylobacter, Chlamydia, Chlamydophila, Legionella* and *Pasteurella*. HER2 positive BC was represented by *Streptococcus* and triple negative BC by *Aerococcus, Arcobacter, Geobacillus, Orientia* and *Rothiawere* [16].

Moreover, species known to infect humans (*Mycobacterium fortuitum* and *Mycobacterium phlei)* were found to be differentially abundant in the tumour samples. In the same study, an increased presence of *Actinobacteria* in the non-cancerous adjacent tissue samples, increase of *Proteobacteria* in tumour tissues and differential abundance for *Cornebacterium, Corynebacterium, Bacillus* and the *Enterobaceriaceae* (*E. coli* and *Salmonella enterica*) was observed, in concordance with other studies [17]. Urbaniak et al. [14]. found abundances of *Prevotella, Lactococcus*, *Streptococcus, Corynebacterium* and *Micrococcus* in healthy patients and *Bacillus, Staphylococcus, Enterobacteriaceae, Comamondaceae* and *Bacteroidetes* in cancer patients. In addition, *Enterobacteriaceae* were found to be relatively more abundant in cancer patients than in healthy controls [14].

In a comparison of published data with our study, we found the same most dominant phyla to be present in breast tissue—mainly *Proteobacteria* and *Actinobacteria*, and to some extent also *Firmicutes* and *Bacteroidetes*. In the results of differential abundance analysis, comparing normal and cancerous tissue, we found some similarities with previous studies. For example, *Methylobacterium, Sphingomonas* and *Bifidobacterium* were reported here and also in previous studies. However, previous studies did not have clear consensus on which bacteria are overrepresented or underrepresented and our study does not claim to bring completely identical observations to any of them.

To mention the limitations of our study, there is also a possibility, that results were influenced by other conditions. For example, age (breast cancer patients from our study are in age 27–79, with average age 59, with most of them over age 50). Healthy women, which were donors of normal breast tissue were indeed younger than women with breast cancer (39–50, with average age of 43.6). According to comparison of microbial content between younger and older donors, we assume results of tumour and normal tissue comparison cannot be assigned to age differences between these groups, since different taxa were correlated with cancer compared to higher age. This analysis was done on Chinese data only since Slovak healthy controls were represented only by very limited number of donors. All Slovak donors were Caucasian women, so ethnicity differences shouldn’t influence the study. For Chinese data, we had just triple negative breast cancer molecular subtype, while Slovak samples were a mix of various subtypes, what might be the reason for some differences between them. Results of clinical traits comparisons has not yet been validated by experimental methods. Relatively small sample size is a disadvantage, since variation between individual samples could possibly influence the results. Biological validation will be needed to prove novel results of this study.

## 4. Materials and Methods

### 4.1. Study Patients

This study included 18 primary breast cancer patients (stage I–III) treated with surgery from April 2012 to February 2015, for whom tumour fresh frozen tissue was available in the biobank. This study represents a substudy of a translational trial that aimed to evaluate the prognostic value of circulating tumour cells in primary breast cancer [35]. Study eligibility criteria and study details were described previously. The study was approved by the Institutional Review Board (IRB) of the National Cancer Institute of Slovakia (TRUSK002, 20.6.2011). Each participant provided signed informed consent before study enrollment.

Healthy donors (*n* = 5) were women undergoing breast surgery for non-cancer related indication (breast cosmesis surgery) without breast cancer who were recruited and consented according to the IRB-approved protocol. Each donor participant signed informed consent.

### 4.2. Tumour Pathology

Pathology review was conducted at the Department of Pathology, National Cancer Institute, Bratislava, Slovakia. Results of hormone receptors, HER2 status and protein p53 were reported either positive or negative on histopathology report without further quantification. Ki-67 labelling index was reported as a percentage of cells with Ki-67 positive nuclear immunostaining. Hormone receptor status was defined as positive for either oestrogen receptor or progesterone receptor vs negative for both; 1% of cells positive for hormone receptor was used as the cut-off to define hormone receptor positivity and HER2 status (normal or amplified). These characteristics of tumours/patients are specified in Table 1.

### 4.3. Additional (Database-Downloaded) Samples

Additional datasets to validate and compare our results were downloaded from NCBI database SRA. Data from study PRJNA553096 appeared to be suitable for our purpose, although of different geographical origin (submitted by Sichuan University, China) [36]. RNA-seq transcriptomic data were obtained from triple-negative breast cancer patients (73 samples) and healthy donors (18 samples). Samples originated from Frozen Primary Tumour Tissue, sequenced by Illumina sequencing technology. Donors had various age (32–80) with an average age of 51, while normal tissue donors had average age of 53 and breast cancer donors of 50.7.

### 4.4. CTC Status Detection

CTC detection was performed as described in full details previously [37]. Briefly, CTCs were detected in peripheral blood by a reverse transcription quantitative PCR (RT-qPCR) based assay utilising cells after CD45 depletion using the RossetteSep kit (Stem Cell Technologies, Vancouver, BC, Canada). For storage at −80 °C and subsequent RNA extraction TRIzolVR LS Reagent (Invitrogen Corporation, Carlsbad, CA, USA) were used. To detect EMT-inducing TF gene transcription (TWIST, SNAI1, SLUG and ZEB1) commercially available TaqMan assays were purchased from Life Technologies Corporation, Pleasanton, CA, USA. The level of transcription was quantified using the delta-Ct method (2^(Ct target—Ct GAPDH)) and values were compared to cut-off values gained from analyses of healthy controls.

### 4.5. RNA Sequencing

#### 4.5.1. Sample Preparation

Fresh-frozen tumour tissue samples were obtained from 5 healthy donors and 18 breast cancer patients at National Cancer Institute (Bratislava, Slovakia). After surgery all tissues were cut into smaller pieces (~500 mg) and placed into a liquid nitrogen tank for storage. Before RNA extraction, tissue samples were ground to a homogenous powder in liquid nitrogen and immediately mixed with 700 ul of DNA/RNA Shield (Zymo Research, Irvine, CA, USA).

#### 4.5.2. Total RNA Extraction

Total RNA was extracted using Quick DNA/RNA Miniprep Plus kit (Zymo Research, Irvine, CA, USA) following the manufacturer’s instructions. RNA concentration was measured using the NanoDrop1000 (Thermo Fisher Scientific, Madison, WI, USA) and RNA Broad range Assays on Qubit fluorometer (Invitrogen, Thermo Fisher Scientific, Carlsbad, CA, USA). Samples that didn’t have sufficient RNA concentration for downstream analyses were concentrated using RNA Clean and Concentrator—5 kit (Zymo Research, Irvine, CA, USA).

#### 4.5.3. rRNA Depletion

For rRNA depletion Ribogone-Mammalian kit (Takara Bio, Mountain View, CA, USA) and manufacturer’s original protocol were used.

#### 4.5.4. cDNA Library Preparation and RNA Sequencing

cDNA libraries were constructed using the SMARTer Stranded RNA-Seq Kit (Takara Bio, San Jose, CA, USA) according to the manufacturer’s instructions. Briefly, protocol started from ~10 ng of rRNA-depleted RNA, which was fragmented and converted to single-stranded cDNA. For cleanup and subsequent (on beads) amplification, AMPure XP beads (Beckman Coulter, Brea, CA, USA) were used. Amplified RNA-seq library was purified by immobilising SPRI beads and washing with 80% ethanol. Library quality control was performed using the Agilent 2100 Bioanalyzer and High Sensitivity DNA Kit (Agilent Technologies, Waldbronn, Germany). For library quantification the Qubit dsDNA HS Assay kit (Invitrogen, Thermo Fisher Scientific, USA) was used. Final RNA-seq libraries were pooled (8 libraries/pool) and sequenced using paired-end sequencing (2 × 75) with NextSeq 500/550 High Output Kit v2.5 (150 Cycles) (Illumina, San Diego, CA, USA) on Illumina NextSeq 550 instrument (Illumina, San Diego, CA, USA).

### 4.6. Quality Control and Data Preparation for Analysis

Paired-end sequencing data were subjected to quality control by FastQC in Galaxy environment [38]. Reads were processed by Trimmomatic [39]. Parameters were chosen according to FastQC results. It was run with the initial ILLUMINACLIP step, with standard adapter sequences (TruSeq2), while settings of this step were set by default. We performed multiple trimming operations: 1. Sliding window trimming (Number of bases to average across—4, Average quality required—22), 2. Cut bases off the start of a read, if below threshold quality (Minimum quality required to keep a base—22), 3. Cut bases off the end of a read, if below threshold quality (Minimum quality required to keep a base—22), 4. Cut 15 bases from start by HEADCROP option. Reads bellow 50 nt length were discarded later.

Data downloaded from SRA were trimmed with the following parameters: Illumina clip step—yes (trueseq3 for hiseq), headcrop 9, leading 22, avgqual 22. Reads bellow 50 nt length were discarded subsequently.

Reads, which passed trimming conditions, were used for subsequent mapping to remove human reads for better performance of microbiome classification. They were mapped on human genome hg38 by BWA for short reads in Galaxy [40], with default options for simple Illumina mode. Unmapped reads were extracted by Samtools view [41] (require that these flags are set—Read is unmapped, Mate is unmapped, Exclude reads with any of the following flags set—read is mapped in proper pair) and converted to fastq format by SamToFastq in Galaxy [42].

### 4.7. Identification and Quantification of the Microbiome: Kraken2

Reads that did not map to the human genome and were longer than 50 nt were used for metatranscriptomic analysis. For the identification of microbes, Kraken2 (Galaxy Version 2.1.1, John Hopkins Universtiy, Center for Computational Biology, Baltimore, Maryland, USA) in Galaxy environment was used [43]. It was used for paired-end data, with minimum base quality set on 20 (which appeared to upgrade specificity), minimum hit groups parameter was set on 2 and standard database (10-Mar-2021, with k-mer length = 35, minimiser length = 31, minimiser spaces = 6) was used. For easier manipulation in later steps, report was made in mpa style. Krona pie chart tool in Galaxy (version 2.7.1, National Biodefense Analysis and Countermeasures Center, MD, USA) was used for visualisation of Kraken2 results [44].

### 4.8. Identification and Quantification of Microbiome: Metaphlan3

For the identification of viral sequences, we used Metaphlan3 software (version 3.0, Department CIBIO, University of Trento, Italy; Harvard T. H. Chan School of Public Health, Boston, MA, USA; The Broad Institute of MIT and Harvard, Cambridge, MA, USA; IEO, European Institute of Oncology IRCCS, Milan, Italy) [45]. Reads that did not map to the human genome were used for further analysis by Metaphlan3, run on Linux command line. Data were analysed as paired-end. Options were set to work only with reads at least 50 nt long, -t parameter were set as “rel_ab_w” to produce the number of identified reads and option to count viruses were added.

### 4.9. Comparison of Microbial Content in Different Disease Status

Statistical analysis, which was done to uncover overrepresented or underrepresented taxa in specified phenotypic groups, was done by LEfSe Galaxy instance (version 1.0, Harvard School of Public Health, Boston, MA, USA) [46,47]. Comparisons were made always between two groups. Using our own data, we compared 18 breast cancer samples (primary tumour) against healthy controls (normal breast tissue), of them 9 samples originated from patients with positive CTC status against 9 CTC negative samples. The numbers of patients with specific markers are specified in Table 1. Parameters for all used LEfSe tools were always set to defaults. Values of Kraken2-computed microbial counts (numbers of fragments assigned to the clade rooted at a taxon) were used to compare the amounts of microbes.

Moreover, additional datasets downloaded from NCBI database SRA (data from study PRJNA553096, submitted by Sichuan University, Chengdu, China) were analysed [36]. First, we compared 19 breast cancer samples (primary tumour) against 18 healthy controls (normal breast tissue), then, to use the full potential of data available and observe difference compared to the use of fewer samples, we compared 73 patient samples against 18 healthy controls.

### 4.10. Pipeline Validation

To identify the rate of false negative results originated from human reads we constructed simulated reads from human genome hg38, with different lengths. To compare with our samples, we sampled fastq files with 65 nt long pair-end reads (corresponding with the length of our sequencing reads after trimming). To see how is read length influencing results, we also sampled fastq file (pair-end) with various length from 20 nt to 150 and also variable lengths with an average of 60 (reads longer than 40 nt produced already good results). For this purpose, we used ART Illumina software in Galaxy (Version 2014.11.03.0, Biostatistics Branch, National Institute of Environmental Health Sciences, Research Triangle Park, NC, USA) [48]. Metaphlan3 and Kraken2 were tested on these data. We also tested Metaphlan3 with reads mapped on human genome hg38 and observing the effect of minimal read length parameter on the results, which were significant and reads shorter than 25 nt showed to be insufficient for analysis.

As a positive control we constructed RNA-seq datasets from microbial reads. For first artificial dataset, we downloaded genomes of 19 microorganisms (Table A1) from NCBI database [49]. Synthetic transcriptomic reads were simulated to be in the same length as our own datasets. We ran Kraken2 and Metaphlan3 with simulated data to see which bacteria were called or missed. As a second positive control, we sampled another simulated RNA-seq dataset, in this case, from viral genomes. We downloaded genomes of 10 randomly chosen viruses from NCBI database (Table A2) [49]. Synthetic transcriptomic reads were simulated to be in the same length as our own datasets. Same, as previously we ran Kraken2 and Metaphlan3 with simulated data to see which viruses were called or misse.

## 5. Conclusions

In this study, we inspected the microbial composition of normal breast tissue and tumour tissue of the breast of donors from Slovakia. Then we used dataset from SRA database (originated in China) for the same purpose. The most abundant phyla were in concordance with previous studies *Proteobacteria*, then *Firmicutes* and *Actinobacteria*, in Slovak samples also *Bacteroides.* In the Chinese dataset, *Cyanobacteria* were also common. Breast tumour tissue were different in microbial composition. Normal tissue appears to be richer for microbes, while many microbes were found to be overrepresented there. Differences in microbial compositions were also found when comparing molecular subtypes of disease, CTC status, markers (HR, HER2, p53), proliferation index Ki67, T and N stage of tumour and tumour grading. The reasons and biological relevance of microbial presence and amounts of their transcripts are not clear and additional studies will be needed to understand the influence on breast cancer and to exploit the microbiome for benefit of cancer patients.

## Figures and Tables

**Table 1:** Patient’s characteristics.

	N	%
All patients	18	100.0
**Histology**		
invasive ductal carcinoma	16	88.9
other	2	11.1
**Grade**		
low and intermediate	7	38.9
high grade	10	55.6
unknown	1	5.6
**T stage**		
T1	12	66.7
>T1	6	33.3
**N stage**		
N0	7	38.9
N+	10	55.6
unknown	1	5.6
**Hormone receptor status (cut-off 1%)**		
negative for both	5	27.8
positive for either	13	72.2
**HER2 status**		
negative	14	77.8
positive	4	22.2
**Ki67 status**		
<20%	7	38,9
>20%	11	61,1
**Molecular subtype**		
luminal A	6	33.3
luminal B	5	27.8
HER2+	4	22.2
triple negative (TN)	3	16.7
**P53 status**		
negative	8	44.4
positive	10	55.6

## Data Availability

RNA-seq data used for the analysis were submitted to SRA database (BioProject ID PRJNA751534). Full data produced by analysis can be found on our website http://www.embnet.sk/supp/BC_metatranscriptomics (accessed on 19 August 2021) in the form of LEfSe graphs.

## Supplementary Materials

The following are available online at https://www.mdpi.com/article/10.3390/ijms22169058/s1.

## Appendix A

**Table A1:** Bacterial genomes and their IDs for NCBI, used to construct artificial mix of bacterial transcriptomes to test how accurate are methods we used for taxonomic identification of reads originated from microbial transcripts.

Species	NCBI Sequence ID
Bacillus subtilis	NC_000964.3
Bifidobacterium lemurum	NZ_CP062948.1
Bifidobacterium scardovii	NZ_AP012331.1
Escherichia albertii	NZ_AP014857.1
Escherichia coli	NZ_CP027599.1
Escherichia fergusonii	NZ_CP057657.1
Hymenobacter jejuensis	NZ_CP040896.1
Hymenobacter nivis	NZ_CP029145.1
Hymenobacter swuensis	NZ_CP007145.1
Lactiplantibacillus plantarum	NZ_CP034997.1
Moraxella Bovoculli	NZ_CP011381.2
Pseudomonas aeruginosa	NC_002516.2
Rhodococcus rhodochroum	NZ_LT906450.1
Sphingomonas alpine	NZ_CP061038.1
Sphingomonas sanxanigens	NZ_CP006644.1
Sphingomonas wittichii	NC_009511.1
Staphylococcus aureus	NC_007795.1
Staphylococcus debuckii	NZ_CP033460.1
Staphylococcus schweitzeri	NZ_LR134304

**Table A2:** Viral genomes and their IDs for NCBI, used to construct artificial mix of viral transcriptomes to test how accurate are methods we used for taxonomic identification of reads originated from viral transcripts.

Species	NCBI Sequence ID
Tomato leaf curl Bangalore virus	NC_003891.1
Ictalurid herpesvirus 1 (Channel catfish virus)	NC_001493.2
Tomato yellow margin leaf curl virus	NC_005852.2
Macacine alphaherpesvirus 1 (monkey B virus)	NC_004812.1
Gallid alphaherpesvirus 2 (Marek disease virus type 1)	NC_002229.3
Cercopithecine alphaherpesvirus 9 (Simian varicella virus)	NC_002686.2
Sheldgoose hepatitis B virus (viruses)	NC_005890.1
Infectious bursal disease virus (Gumboro virus)	NC_004178.1
Human alphaherpesvirus 3 (Varicella-zoster virus)	NC_001348.1
SARS-CoV2	NC_045512.2

## References

[1] Goodman B., Gardner H. (2018). The microbiome and cancer. J. Pathol..

[2] Zhu B., Wang X., Li L. (2010). Human gut microbiome: The second genome of human body. Protein Cell.

[3] Turnbaugh P.J., Ley R.E., Hamady M., Fraser-Liggett C.M., Knight R., Gordon J.I. (2007). The human microbiome project. Nature.

[4] Urbaniak C., Cummins J., Brackstone M., Macklaim J.M., Gloor G.B., Baban C.K., Scott L., O’Hanlon D.M., Burton J.P., Francis K.P. (2014). Microbiota of human breast tissue. Appl. Environ. Microbiol..

[5] Xuan C., Shamonki J.M., Chung A., Dinome M.L., Chung M., Sieling P.A., Lee D.J. (2014). Microbial dysbiosis is associated with human breast cancer. PLoS ONE.

[6] Fernández M.F., Reina-Pérez I., Astorga J.M., Rodríguez-Carrillo A., Plaza-Díaz J., Fontana L. (2018). Breast Cancer and Its Relationship with the Microbiota. Int. J. Environ. Res. Public Health.

[7] de Martel C., Ferlay J., Franceschi S., Vignat J., Bray F., Forman D., Plummer M. (2012). Global burden of cancers attributable to infections in 2008: A review and synthetic analysis. Lancet Oncol..

[8] Schlaeppi K., Bulgarelli D. (2015). The plant microbiome at work. Mol. Plant Microbe Interact..

[9] Goodwin S., McPherson J.D., McCombie W.R. (2016). Coming of age: Ten years of next-generation sequencing technologies. Nat. Rev. Genet..

[10] Borchmann S. (2021). An atlas of the tissue and blood metagenome in cancer reveals novel links between bacteria, viruses and cancer. Microbiome.

[11] Hieken T.J., Chen J., Hoskin T.L., Walther-Antonio M., Johnson S., Ramaker S., Xiao J., Radisky D.C., Knutson K.L., Kalari K.R. (2016). The Microbiome of Aseptically Collected Human Breast Tissue in Benign and Malignant Disease. Sci. Rep..

[12] Costantini L., Magno S., Albanese D., Donati C., Molinari R., Filippone A., Masetti R., Merendino N. (2018). Characterization of human breast tissue microbiota from core needle biopsies through the analysis of multi hypervariable 16S-rRNA gene regions. Sci. Rep..

[13] Chadha J., Nandi D., Atri Y., Nag A. (2021). Significance of human microbiome in breast cancer: Tale of an invisible and an invincible. Semin. Cancer Biol..

[14] Urbaniak C., Gloor G.B., Brackstone M., Scott L., Tangney M., Reid G. (2016). The Microbiota of Breast Tissue and Its Association with Breast Cancer. Appl. Environ. Microbiol..

[15] Wang H., y mAltemus J., Niazi F., Green H., Calhoun B.C., Sturgis C., Grobmyer S.R., Eng C. (2017). Breast tissue, oral and urinaricrobiomes in breast cancer. Oncotarget.

[16] Banerjee S., Tian T., Wei Z., Shih N., Feldman M.D., Peck K.N., DeMichele A.M., Alwine J.C., Robertson E.S. (2018). Distinct Microbial Signatures Associated with Different Breast Cancer Types. Front. Microbiol..

[17] Thompson K.J., Ingle J.N., Tang X., Chia N., Jeraldo P.R., Walther-Antonio M.R., Kandimalla K.K., Johnson S., Yao J.Z., Harrington S.C. (2017). A comprehensive analysis of breast cancer microbiota and host gene expression. PLoS ONE.

[18] Kosaka A., Yan H., Ohashi S., Gotoh Y., Sato A., Tsutsui H., Kaisho T., Toda T., Tsuji N.M. (2012). Lactococcus lactis subsp. cremoris FC triggers IFN-γ production from NK and T cells via IL-12 and IL-18. Int. Immunopharmacol..

[19] Baumgarten S.C., Frasor J. (2012). Minireview: Inflammation: An instigator of more aggressive estrogen receptor (ER) positive breast cancers. Mol. Endocrinol..

[20] Flores R., Shi J., Fuhrman B., Xu X., Veenstra T.D., Gail M.H., Gajer P., Ravel J., Goedert J.J. (2012). Fecal microbial determinants of fecal and systemic estrogens and estrogen metabolites: A cross-sectional study. J. Transl. Med..

[21] Flores R., Shi J., Gail M.H., Gajer P., Ravel J., Goedert J.J. (2012). Association of fecal microbial diversity and taxonomy with selected enzymatic functions. PLoS ONE.

[22] Chan A.A., Bashir M., Rivas M.N., Duvall K., Sieling P.A., Pieber T.R., Vaishampayan P.A., Love S.M., Lee D.J. (2016). Characterization of the microbiome of nipple aspirate fluid of breast cancer survivors. Sci. Rep..

[23] Alexander J.L., Wilson I.D., Teare J., Marchesi J.R., Nicholson J.K., Kinross J.M. (2017). Gut microbiota modulation of chemotherapy efficacy and toxicity. Nat. Rev. Gastroenterol. Hepatol..

[24] Chen J., Douglass J., Prasath V., Neace M., Atrchian S., Manjili M.H., Shokouhi S., Habibi M. (2019). The microbiome and breast cancer: A review. Breast Cancer Res. Treat..

[25] Hill A., Hanson M., Bogle M.A., Duvic M. (2004). Severe radiation dermatitis is related to *Staphylococcus aureus*. Am. J. Clin. Oncol..

[26] Garrido-Cardenas J.A., Manzano-Agugliaro F. (2017). The metagenomics worldwide research. Curr. Genet..

[27] Cresci G.A., Bawden E. (2015). Gut Microbiome: What We Do and Don’t Know. Nutr. Clin. Pract..

[28] Banerjee S., Wei Z., Tan F., Peck K.N., Shih N., Feldman M., Rebbeck T.R., Alwine J.C., Robertson E.S. (2015). Distinct microbiological signatures associated with triple negative breast cancer. Sci. Rep..

[29] Shakya M., Lo C.C., Chain P.S.G. (2019). Advances and Challenges in Metatranscriptomic Analysis. Front. Genet..

[30] Lugli G.A., Milani C., Mancabelli L., Turroni F., van Sinderen D., Ventura M. (2019). A microbiome reality check: Limitations of in silico-based metagenomic approaches to study complex bacterial communities. Environ. Microbiol. Rep..

[31] Hadžega D., Karaba M., Minarik G., Benca J., Sedlackova T., Macuch J., Sieberova G., Pindak J., Kalavska K., Klucar L. (2021). Comenius University, Bratislava, Slovakia.

[32] Bush S.J., Connor T.R., Peto T.E.A., Crook D.W., Walker A.S. (2020). Evaluation of methods for detecting human reads in microbial sequencing datasets. Microb. Genom..

[33] Castellarin M., Warren R.L., Freeman J.D., Dreolini L., Krzywinski M., Strauss J., Barnes R., Watson P., Allen-Vercoe E., Moore R.A. (2012). Fusobacterium nucleatum infection is prevalent in human colorectal carcinoma. Genome Res..

[34] Mazmanian S.K., Round J.L., Kasper D.L. (2008). A microbial symbiosis factor prevents intestinal inflammatory disease. Nature.

[35] Mego M., Karaba M., Minarik G., Benca J., Silvia J., Sedlackova T., Manasova D., Kalavska K., Pindak D., Cristofanilli M. (2019). Circulating Tumor Cells with Epithelial-to-mesenchymal Transition Phenotypes Associated With Inferior Outcomes in Primary Breast Cancer. Anticancer Res..

[36] Sequence Read Archive (SRA). https://www.ncbi.nlm.nih.gov/bioproject/PRJNA553096.

[37] Smolkova B., Cierna Z., Kalavska K., Miklikova S., Plava J., Minarik G., Sedlackova T., Cholujova D., Gronesova P., Cihova M. (2020). Increased Stromal Infiltrating Lymphocytes Are Associated with the Risk of Disease Progression in Mesenchymal Circulating Tumor Cell-Positive Primary Breast Cancer Patients. Int. J. Mol. Sci..

[38] Babraham Bioinformatics, FastQC. http://www.bioinformatics.babraham.ac.uk/projects/fastqc/.

[39] Bolger A.M., Lohse M., Usadel B. (2014). Trimmomatic: A flexible trimmer for Illumina sequence data. Bioinformatics.

[40] Li H., Durbin R. (2009). Fast and accurate short read alignment with Burrows-Wheeler transform. Bioinformatics.

[41] Li H., Handsaker B., Wysoker A., Fennell T., Ruan J., Homer N., Marth G., Abecasis G., Durbin R. (2009). The Sequence Alignment/Map format and SAMtools. Bioinformatics.

[42] Picard Broad Institute, GitHub Repository. http://broadinstitute.github.io/picard/.

[43] Wood D.E., Salzberg S.L. (2014). Kraken: Ultrafast metagenomic sequence classification using exact alignments. Genome Biol..

[44] Ondov B.D., Bergman N.H., Phillippy A.M. (2011). Interactive metagenomic visualization in a Web browser. BMC Bioinform..

[45] Beghini F., McIver L.J., Blanco-Míguez A., Dubois L., Asnicar F., Maharjan S., Mailyan A., Manghi P., Scholz M., Thomas A.M. (2021). Integrating taxonomic, functional, and strain-level profiling of diverse microbial communities with bioBakery 3. eLife.

[46] Schloss P.D., Westcott S.L., Ryabin T., Hall J.R., Hartmann M., Hollister E.B., Lesniewski R.A., Oakley B.B., Parks D.H., Robinson C.J. (2009). Introducing mothur: Open-Source, Platform-Independent, Community-Supported Software for Describing and Comparing Microbial Communities. Appl. Environ. Microbiol..

[47] Segata N., Izard J., Waldron L., Gevers D., Miropolsky L., Garrett W.S., Huttenhower C. (2011). Metagenomic biomarker discovery and explanation. Genome Biol..

[48] Huang W., Li L., Myers J.R., Marth G.T. (2011). ART: A next-generation sequencing read simulator. Bioinformatics.

[49] NCBI. https://www.ncbi.nlm.nih.gov/.

