# Supplementary material for: Uncovering Microbial Composition in Human Breast Cancer Primary Tumour Tissue Using Transcriptomic RNA-seq
Source: Int J Mol Sci. 2021 Aug 22;22(16):9058. doi: 10.3390/ijms22169058 (PMC8396677; doi:10.3390/ijms22169058)

Figure S1. Boxplot visualisation of the most frequent taxa prevalence. The graph shows % amounts of bacterial taxa reads out of all bacterial reads in samples of different basic groups. All samples used in the study are included in the graph.

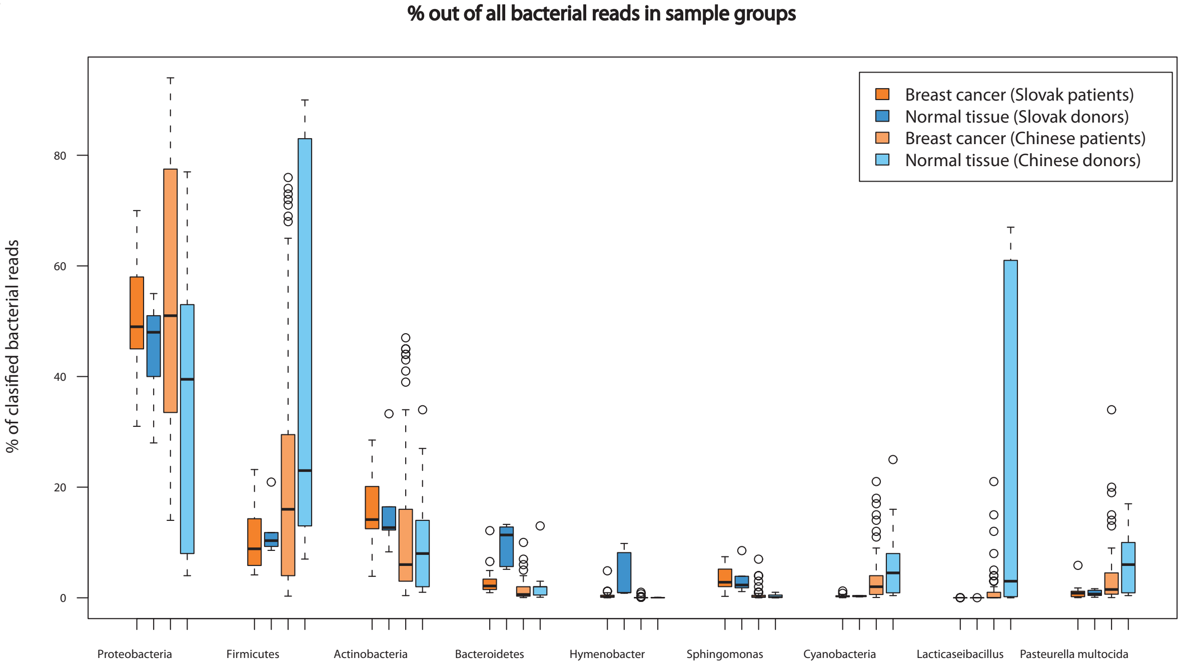

Supplement: Supplementary file 1 [file ijms-22-09058-s001.zip › Figure S1_v2_ps.pdf]
